# The Association between Cultural Competency, Structural Empowerment, and Effective Communication among Nurses in Saudi Arabia: A Cross-Sectional Correlational Study

**DOI:** 10.3390/nursrep12020028

**Published:** 2022-04-08

**Authors:** Rawaih Falatah, Lina Al-Harbi, Eman Alhalal

**Affiliations:** 1 College of Nursing, King Saud University, Riyadh 11451, Saudi Arabia; ealhalal@ksu.edu.sa; 2 King Fahad Hospital Jeddah, Kingdom of Saudi Arabia, Jeddah 23325, Saudi Arabia; lialharbi@moh.gov.sa

**Keywords:** cultural competency, structural empowerment, effective communication, nursing workforce, Saudi Arabia

## Abstract

This study aimed to examine the association between cultural competency, structural empowerment, and effective communication among nurses in Saudi Arabia. A cross-sectional correlational design was used. The study questionnaire utilized three scales: the Culture Competence Scale, Conditions for Work Effectiveness Questionnaire-II, and Communication Competency Assessment Scale. All the scales were culturally adapted and translated using an integrated method. The questionnaire was distributed through an online survey using a convenience sampling approach. Data were collected from 396 participants. The findings showed statistically significant association between cultural competency and effective communication (r = 0.747, *p* < 0.001) and between structural empowerment and cultural competency (r = −0.123, *p* = 0.014). Moreover, the overall model with effective communication and structural empowerment as predictors, controlling for nurses’ nationality significantly explains 56% of the variance in cultural competency. Structural empowerment did not significantly predict cultural competency (b = −0.052, β = −0.069, *p* < 0.052, 95% CI = [−0.104, −0.001]), while effective communication was found to be a significant positive independent predictor of cultural competency (b = 0.745, β = 0.741, *p* < 0.001, 95% CI = [0.677, 0.811]). The findings underline the need to make effective communication courses mandatory in undergraduate nursing curricula. Healthcare systems should be built such that they support the empowerment of the nursing workforce from different nationalities and establish effective communication policies to enhance cultural competency among nurses. Future research in this area is needed to validate the result of this study.

## 1. Introduction

Culture comprises a shared system of norms, meanings, values, and beliefs, which directly influence workplace behaviors and attitudes [1]. Within an organization, cultural differences can be identified as having groups that hold different values, beliefs, and linguistic backgrounds, including different generations and genders [2]. Global nursing workforce mobility is well documented across the nursing literature. In Saudi Arabia, in particular, the nursing workforce has different nationalities, leading to a culturally different environment [3]. Therefore, there is a need for cultural competency [4], which is defined as a process in which the nurse strives to work effectively within the cultural context of an individual who comes from a different cultural background [5]. It increases the awareness of nurses to reevaluate their culture impact on their practice [6]. Culturally competent nurses understand that each person is unique in identifying health and illness according to their beliefs. Therefore, while working, nurses need to be aware and knowledgeable about the different cultures in their work environment [7]. Nursing leaders should understand that culturally diverse work environments can enhance organization effectiveness, but it can also lead to ineffective organizations [3,5,8]. If cultural differences are not handled effectively, they could be the underlying reason for communication issues, which may eventually affect patient safety [1]. 

Enhancing cultural competence can significantly decrease stereotyping and time pressure, as well as distress among nurses. Therefore, cultural competence education is strongly recommended to be started early to prepare nurses with appropriate culturally competent care and equip them to become effective team members and provide comprehensive patient-centered care [9,10]. Various techniques and strategies are used by healthcare leaders and managers to enhance cultural competency among nurses [9,11,12]. Among these practices is enhancing nurses’ involvement and willingness to improve performance through structural empowerment [13]. 

Structural empowerment is defined as the degree an organization has reached in providing access to their staff toward opportunity, information, resources, support, and power [14]. It is known to be an approach to increase nurses’ accountability and commitment, which could be achieved by giving opportunities for nurses to advance their professional competency level with continuous support. As explained by Lee and Kim [13], structural empowerment promotes the importance of employees’ participation and interaction with organization members. Empowering staff with better work conditions can align the nurses’ career development goals with organization goals leading to the growth and development of the organization [15]. Furthermore, structural empowerment can improve staff competency resulting in enhanced workplace dynamics, effective workplace communication, and improved patient care outcomes, which facilitate the accomplishment of the healthcare organization’s goals and care quality standards [4,16,17]. Indeed, structural empowerment was also found to support effective communication in healthcare organizations [13].

Handling culturally diverse teams is not only about different communication styles, but also under the scope of cultural communication, including direct versus indirect communication, accent and fluency issues, different attitudes toward hierarchy and authority, and conflicting norms leading to different decision making [18]. Thus, effective communication can be defined as the situation wherein communication processes lead to a proper understanding of the message without interruption, and it includes asking questions for clarification and expression of opinions by both speaker and listener; the outcome of effective communication should be all parties (sender and receiver) reaching mutual understanding in the process [19]. Effective communication practices can lead to the achievement of organizational goals, and it is highly integrated with developing cultural competence skills. It can provide the ability to consider different beliefs and values and use effective methods in planning and providing care [20]. Thus, through the practice of effective communication techniques, and the integration of these techniques into organizational policies, cultural competence can be expressed by the employees, which may subsequently lead to an improvement in healthcare quality. 

In the nursing literature, numerous studies have established the association between effective communication and cultural competency. Nonetheless, no known studies have explored the association between structural empowerment, effective communication, and cultural competency. Thus, this study aims to examine the association between cultural competency, structural empowerment, and effective communication among nurses in Saudi Arabia. 

According to the Kanter theory of structural empowerment [21], power in organizations originates from structural factors in the workplace. Kanter distinguishes between two sorts of power: formal and informal. Formal power is found in high-profile occupations that allow for decision-making freedom; informal power is gained via alliances with individuals at all levels within and beyond the organization: superiors, colleagues, and others. The power structure relates to the organization’s information, support, and resources lines. According to Kanter’s idea, empowered workers have more access to workplace empowerment systems and are hence more productive. Kanter’s empowerment theory was expanded to include the impact of structural empowerment on work outcomes, such as work satisfaction [22], perceived respect [23], and knowledge transfer [24]. Thus, we propose based on Kanter’s theory and previous studies that structural empowerment is associated with effective communication and higher cultural competence among nurses. Particularly, we examined the following hypotheses:

**Hypothesis** **1** **(H1).** *Structural empowerment significantly predicts effective communication.*

**Hypothesis** **2** **(H2).** *Effective communication and structural empowerment significantly predict cultural competency controlling for nationality.*

## 2. Materials and Methods

### 2.1. Research Design and Setting

A cross-sectional descriptive correlational design was utilized. The study was conducted in three hospitals in Jeddah City, Saudi Arabia. The first hospital is located in the center of the city with a capacity of 700 beds and a total number of 1200 nurses. It is one of the main tertiary hospitals in Jeddah that is specialized in adult care services only. The second hospital is a tertiary hospital located on the east side of the city with 300 beds and provides adult, maternity, and pediatric healthcare services. The total number of nurses in this hospital is 700 nurses. The third hospital is a tertiary hospital located on the north side of the city. It has a capacity of 500 beds and provides adult, maternity, and pediatric healthcare services. The total number of nurses in the third hospital is 1000 nurses.

### 2.2. Participants

A nonprobability convenience sampling approach was used to recruit the nurses. The inclusion criteria were a registered nurse and working in one of the three hospital settings for more than six months. The sample size was established using an effect size of 0.1, an alpha level of 0.05 and a power of 0.8, and 3 predictors by G* power; 81 registered nurses were needed.

### 2.3. Data Collection

We collected the data using a questionnaire that contains four parts: (1) sociodemographic, (2) culture competency, (3) condition for work effectiveness-II (CWEQ-II), which measures structural empowerment, and (4) communication competency. All the used scales and demographic questions were entered into an online survey in both English and Arabic. Data collection started on the 27th of September 2020 and ended on the 28th of November 2020. Due to COVID-19 pandemic restriction, the nursing offices requested the questioners to use an online survey to decrease the exchange of papers between the study participants, nursing office staff, and researchers. The created online survey link was sent to the nursing office in each hospital. After that, the nursing office sent the link to the staff nurses who meet the inclusion criteria. The link was sent again as a reminder after one month. Distributing the survey through the official email to all eligible staff helped in minimizing the selection bias. In addition, nurses are an internet-literate population, and this also minimizes the selection bias. Nonetheless, the response rate was 13.6%, and this low response rate could be explained by the fact that COVID-19 pandemic has impacted the nurses’ participation in this study due to higher workload.

#### 2.3.1. Scales’ Cultural Adaptation and Translation

All the scales were culturally adapted and translated using the integrated method developed by Sidani et al. [25]. First, the conceptual equivalence of the scales was evaluated in collaboration with five bicultural and bilingual nurses. The nurses were asked to score each scale item separately in terms of understanding and context relevance (10-point numeric rating scale with extremes of ‘not at all’ and ‘very much’). The items were mostly assessed as simple to comprehend (comprehension > 5) and relevant (relevance > 5). As a result, the content validity index (CVI) varied between 0.9 and 1. Professional forward translators then translated the scales into Arabic. Back translation, however, was not done because it was an optional step in the approach that was used. The final version of the modified and translated measures was tested on 30 nurses in a pilot study. The reliability coefficient alpha for Culture Competency scale, Conditions for Work Effectiveness Questioner-II, and Communication Competency Assessment Scale was 0.83, 0.92, and 0.93, respectively. These Cronbach’s alpha scores indicated a high level of scale reliability. All questionnaires’ items were asked in two languages, Arabic and English.

#### 2.3.2. Sociodemographic Information

Based on the reviewed literature, we asked the participants about six sociodemographic variables, which are gender, years of experience, setting (hospital), nationality (Saudi or non-Saudi), and qualifications.

#### 2.3.3. Culture Competency

The Cultural Competence Scale [26] was used to measure the participants’ cultural competency level after obtaining the scale author’s approval. The scale consists of 20 items measured on a five-point Likert scale: strongly agree (1), agree (2), neutral (3), disagree (4), and strongly disagree (5). Averaging was used to calculate the scale score. The scale was used in the nursing studies with the test-re-test reliability of (Rho = 0·97), and the internal consistency value of the scale was 0.86 [26,27].

#### 2.3.4. Structural Empowerment

CWEQ-II [22] was used to measure structural empowerment after obtaining approval. The scale contains 21 items under seven subscales assessed on five-point Likert scales: opportunity (three items), information access (three items), support access (three items), resources access (three items), work setting (three items), activities opportunity (four items), and kind of opportunity (two items). Averaging is used to calculate total scale and subscale scores. The scale was previously used in the nursing literature and the Cronbach’s α coefficient of the scale was 0.83 [28].

#### 2.3.5. Communication Competency

The Communication Competency Assessment Scale [29] was used to measure participants’ communication effectiveness. The approval to use the scale was obtained from Texas Agricultural and Mechanical College. It is composed of 23 items under five subscales: communication process (five items), communication styles (five items), communication through conflict (five items), feedback in communication (four items), and cultural barriers in communication (four items). The items are measured on a five-point Likert scale: strongly agree (1), agree (2), neutral (3), disagree (4), and strongly disagree (5), and averaging is used to calculate the total scale and subscale scores. The internal consistency reliability of the scale was not reported in the literature, nor was it used among nurses; thus, it was tested in a pilot study. The result of the reliability test (α = 0.93) allowed the use of the scale in this study.

### 2.4. Data Analysis

The analysis was conducted using IBM SPSS statistics version 27.0.1 (Armonk, NY, US). In this step, data cleaning was performed, and missing data were analyzed using missing data analysis. Series means were used for each variable containing missing data. Out-of-range values were analyzed, and outlier detection was done; neither returnedany out of range findings. Variables scoring for all the scales was done based on the original scales’ instructions by averaging. We did not use cut-off points or medina splitting because it was not instructed by the scales’ developers. Univariate descriptive analysis was performed using percentage, mean, range, and standard deviation. Bivariate analysis was conducted using both Pearson and Spearman correlations. Multiple regression analysis was performed to examine the predictability of cultural competency through structural empowerment and effective communication. Based on the bivariate analysis, we did not include any of the demographic variables as a confounding variable in the regression model.

### 2.5. Ethical Considerations

Approval was obtained from the ethical review board of the Saudi Ministry of Health (H-02-J-002). The approval, along with an official permission from the hospital’s director, were sent to the nursing department in the three selected hospitals to encourage the staff to participate in the study. The participants were oriented on their voluntary participation and the assurance of their anonymity through the provision of informed consent at the beginning of each online survey that began with consent through two choices: agree or disagree. The participants were informed that the data would be released for research purposes in an aggregated format. In addition, the data was stored in a password-protected drive that could only be accessed by the research team.

## 3. Results

A total of 396 nurses participated in the study. The participants’ sociodemographic characteristics are shown in Table 1. The majority of the participants were female (92.5%). Most of the participants held a Bachelor of Science Nursing degree (*n* = 270, 69.4%), whereas 27.8% (*n* =108) held a diploma in nursing degree. Over 50% reported their nationality as Saudi, while 47.8% (*n* = 186) reported their nationality as non-Saudi. The average years of experience was 6.75 (SD = 5.14) and the range of the years of experience was found to be 29 years (1 to 30 years).

The average cultural competency among the study participants was 2.03 (SD = 0.46, range = 3). The overall condition for work effectiveness-II scale score was 2.96 (SD = 0.62, range = 3.81). The highest average among condition for work effectiveness-II subscales was opportunity ($\bar{x}$ = 3.24, SD = 0.97, range = 4) and the lowest was kind of opportunity ($\bar{x}$ = 2.60, SD = 0.76, range = 4). The average score for information access was 3.10 (SD = 0.90, range = 4), support access was 3.00 (SD = 0.89, range = 4), resources access was 2.95 (SD = 0.83, range = 4), work setting was 2.83 (SD = 0.92, range = 4), and activities opportunity was 2.98 (SD = 0.81, range = 4). Finally, the overall communication competency score was 1.870 (SD = 0.46, range = 3.00). The highest average among communication competency assessment subscales was communication styles ($\bar{x}$ = 1.94, SD = 0.50, range = 3), whereas the lowest average was feedback in communication ($\bar{x}$ = 1.82, SD = 0.55, range = 3.75). The average score for communication process competency among the study participants was 1.88 (SD = 0.46, range = 3.00), communication through conflict was 1.85 (SD = 0.54, range = 3.80), cultural barriers in communication was 1.86 (SD = 0.54, range = 3.00). 

### 3.1. The Association between the Sociodemographic Variables and the Study Variables

Both Pearson and Point-Biserial correlation was employed to investigate the relationship between the sociodemographic variables and study variables. Nurses’ nationality was the only sociodemographic variable that has statistically significant association with one of the study variables, which is structural empowerment (r_pb_ = 0.296, *p* < 0.001). Among the study variables, the correlation results showed statistically significant positive association between effective communication and culture competency (r = 0.747, *p* < 0.001). The correlation finding, on the other hand, revealed statistically significant negative association between structural empowerment and cultural competency (r = −0.123, *p* = 0.014). Thus, the increase in effective communication leads to an increase in the participants’ culture competency. However, culture competency will decrease as a result of the increase in structural empowerment.

### 3.2. Mean Difference in the Study Variables Based on Participants’ Nationality

Based on the association between structural empowerment and participants’ nationality, the mean difference in structural empowerment based on the participants nationality was examined using an independent samples *t*-test. The results revealed that based on the participants’ nationality, there was statistically significant mean difference in all of the subscales of the measure of structural empowerment as follows: opportunity (t = −3.617, *p* < 0.000), information access (t = −5.605, *p* < 0.000), support access (t = −6.707, *p* < 0.000), resources access (t = −4.339, *p* < 0.000), work setting (t = −5.955, *p* < 0.000), activities opportunity (t = −5.148, *p* < 0.000), and kind of opportunity (t = 1.970, *p* < 0.05). It was noted that the non-Saudis mean was statistically significantly higher in all of the previously mentioned structural empowerment component, but for the kind of opportunity dimension. Additionally, there was a statistically significant mean difference based on the nationality on and overall conditions for work effectiveness-II score (t = −6.148, *p* < 0.000). 

### 3.3. Results According to the Study Hypothesis

The hypothesis was tested using multiple regression to examine whether structural empowerment predicts effective communication. The model was not statistically significant (F (2, 39) = 3.435, *p* = 0.065). Structural empowerment predicted 0.9% of the change in effective communication (b = −0.069, β = −0.093, *p* = 0.065, 95% CI = (−0.143, 0.004)). Based on the result it could be concluded that the hypothesis was not supported.

The hypothesis was tested using multiple regression to examine whether effective communication and structural empowerment predict cultural competency controlling for participants’ nationality. Only nationality was included as a controlling variable based on the bivariate analysis result. First, nationality was entered and then structural empowerment and effective communication were entered. The first model was statically insignificant (F (1, 387) = 0.245, *p* = 0.621), and it predicted 0.1 % of the change in cultural competency. When we added structural empowerment and effective communication, the model became statistically significant (F (3, 385) = 165.129, *p* < 0.001), and overall, the model predicted 56% of the change in nurses’ cultural competency (R^2^ = 0.75). Structural empowerment was found to be an insignificant predictor of cultural competency (b = −0.052, β = −0.069, *p* < 0.052, 95% CI = [−0.104, −0.001]). Effective communication was found to be a significant positive predictor of cultural competency (b = 0.745, β = 0.741, *p* < 0.001, 95% CI = [−0.677, 0.811]). Thus, the result revealed that as effective communication increases, cultural competency increases. This result supports the conclusion that the model of structural empowerment and effective communication significantly predict cultural competency.

## 4. Discussion

This study aimed to address a gap in the literature by examining the association between cultural competency, structural empowerment, and effective communication among nurses in Saudi Arabia. This is the first study that examines the association between these variables among nurses in Saudi Arabia. The study results showed that structural empowerment and effective communication significantly predicted cultural competency.

First, in this study, structural empowerment did not significantly predict effective communication. This finding contradicts the finding from the study conducted by Armellino et al. [30], which found a positive association between structural empowerment and each of communication openness, feedback, and communication about errors. Additionally, structural empowerment was found to be significantly positively associated with newly graduated nurses’ ability to speak up and communicate in cases of unsafe practices [31]. The current study result might be due to having around 50% of the sample as non-Saudi nurses. Almutairi et al. [32] argued that among the consequences of hiring non-Saudi nurses is suboptimal communication and short periods of employment that might impact the role of these nurses in the healthcare system. Indeed, the structural empowerment of non-Saudi nurses might be lower than Saudi nurses.

The finding revealed that structural empowerment did not predicts nurses’ cultural competency. Although being insignificant, the negative direction of this association needs further investigation. Furthermore, in the literature, there are no studies that examined the association between structural empowerment and cultural competency among nurses in Saudi Arabia; nonetheless, this result might be comparable with the positive correlation between structural empowerment and interpersonal conflict found among a sample of multinational nurses working in Pakistan [14].

This study showed that effective communication and structural empowerment significantly predicted cultural competence. Practicing effective communication was included in several studies aiming to enhance cultural competence or to develop cultural competence education or training programs and was even included as one of the guidelines for cultural competence [33,34,35]. Structural empowerment access was included in cultural competence studies as a work opportunity for increasing knowledge and skills [3,4,5,6,9,10]. In the current study, there was a significant mean difference in all the dimensions of structural empowerment based on the participants’ nationality. Thus, we included nationality in the model as a confounding variable. Further research is needed to validate the impact of nationality on structural empowerment and its association with other organizations, staff, and patients’ outcomes.

### 4.1. Implication

The nursing profession in Saudi Arabia is continuously growing and moving forward [34,36,37,38]. Therefore, including new courses and programs is preferred to enhance the nursing field knowledge and skills. The present results provide clear evidence on the importance of practicing effective communication; therefore, nursing communication courses are advised to be included in the nursing curriculum as one of the main courses. Additionally, cultural competence awareness needs to be enhanced starting from the undergraduate nursing education.

The nursing workforce in Saudi Arabia is one of the most highly diverse nursing workforces internationally [3,5,6,11]; intercultural communication theory should be acknowledged as one of the main nursing theories in undergraduate and postgraduate nursing programs. Furthermore, including cultural competence and communication competence in the essential healthcare organization safety standards would be preferred for maintaining healthcare quality and safety [39]. Thus, cultural competence and effective communication awareness and training should be included in health institute orientation programs and ongoing educational in-services.

This study is the first to examine the relationship between cultural competence, conditions for work effectiveness, and effective communication in Saudi Arabia; additional similar studies are required to confirm and strengthen the study results. This study included only government hospitals; future studies should also consider private medical facilities. In addition, other healthcare specialties should also be included.

### 4.2. Limitation

The study has several limitations. A convenience sampling technique was applied, which limits the generalizability of the findings. The study was conducted at government hospitals in the Jeddah region only. In addition, the number of male participants in the study was limited; thus, the findings based on the participants’ gender are not conclusive. The study did not include participant age in the demographic data as it was replaced by the participant’s years of experience. Nevertheless, Alharbi et al. [38] found no statistically significant association between nurses’ age and cultural competence in Saudi Arabia. Finally, the use of self-assessment may affect the study; however, appropriate measures were taken to control the implication of bias.

## 5. Conclusions

In conclusion, the current study showed a significant mean difference based on participants’’ nationality in all the dimensions of structural empowerment. This might lead to the negative association between structural empowerment and cultural competency. Additionally, the association between structural empowerment and effective communication was insignificant. Both of these results need further investigation and validation. The findings underline the need to make effective communication courses mandatory in undergraduate nursing curricula. Healthcare systems should be built such that they support the empowerment of the nursing workforce from different nationalities and establish effective communication policies to enhance cultural competency among nurses.

## Figures and Tables

**Table 1:** Frequency Distributions of the Participants’ Sociodemographic Characteristics (*n* = 396).

Characteristics	Value	Frequency (*n*)	Percentage (%)
Gender	Male	29	7.5
	Female	360	92.5
Qualification	Diploma	108	27.8
	Bachelor’s	270	69.4
	Master’s	11	2.8
Nationality	Saudi	203	52.2
	Non-Saudi	186	47.8
Characteristics	Range	Mean	SD
Experience	29	6.75	5.14

## Data Availability

The data are available by request due to ethical consideration.

## Manuscript Preparation

We confirm that the paper adheres to STROBE reporting guidelines for cross-sectional studies.
